# The mediating effect of quality of life between family support and advance care planning readiness of older adult patients with chronic diseases

**DOI:** 10.3389/fpubh.2025.1619449

**Published:** 2025-07-21

**Authors:** Yongli Lyu, Yafang Liu, Yuechen Liu, Xinlian Yang, Kai Guo

**Affiliations:** ^1^Union Hospital, Tongji Medical College, Huazhong University of Science and Technology, Wuhan, China; ^2^School of Education, Central China Normal University, Wuhan, China; ^3^School of Nursing, Tongji Medical College, Huazhong University of Science and Technology, Wuhan, China; ^4^Tongji Hospital, Tongji Medical College, Huazhong University of Science and Technology, Wuhan, China; ^5^Department of Pediatrics, Huangshi Central Hospital, Affiliated Hospital of Hubei Polytechnic University, Huangshi, China

**Keywords:** older adult chronic diseases, family support, quality of life, advance care planning, mediating effect

## Abstract

**Objective:**

To investigate the mediating effect of quality of life (QoL) between family support and advance care planning (ACP) readiness among older adult patients with chronic diseases, thereby providing evidence for nursing administrators to strengthen family support and promote ACP preparedness.

**Methods:**

From November 2024 to February 2025, a total of 262 older adult patients with chronic diseases were recruited via convenience sampling from seven tertiary hospitals located in Guangdong, Hainan, Sichuan, Heilongjiang, and Hubei provinces. Data were collected using a general information questionnaire, the EuroQoL-5 Dimension (EQ-5D-5L) scale, the Family Support Scale, and the Advance Care Planning Readiness Scale.

**Results:**

The median EQ-5D-5L utility score was 0.831 (IQR: 0.640–0.942), the family support score was 12.00 (IQR: 12.00–13.00), and the mean ACP readiness score was 81.97 ± 9.88. Family support was found to be positively correlated with ACP readiness (*r* = 0.515, *p* < 0.01). Similarly, quality of life was also positively associated with ACP readiness (*r* = 0.552, *p* < 0.01). Furthermore, family support exhibited positive correlation with quality of life (*r* = 0.403, *p* < 0.01). Mediation analysis indicated that quality of life partially mediated the interrelationship between family support and ACP readiness, accounting for 25.5% of the total effect.

**Conclusion:**

Quality of life plays a mediating role between family support and ACP readiness in older adult patients with chronic diseases. Nursing administrators should prioritize improving quality of life and fostering supportive family environments to enhance ACP preparedness.

## 1 Introduction

Advance care planning (ACP) (1) refers to a process in which patients, who are still conscious and capable of making decisions, engage in discussions with family members and medical staff about future medical choices in the event of serious illness. In China, advance directives (ADs) are often referred to as “living wills,” which are legally effective oral or written document where an individual expresses their wishes for treatment preferences and designates a healthcare proxy. ADs are the tangible outcomes of ACP discussions between patients, physicians, and family members (2). The focus of ACP implementation is to assist patients in clarifying their personal values and goals for future medical care, and to facilitate in-depth communication among family members, healthcare providers, and medical decision-makers. This process enables patients and their families to prepare for future medical decisions in the event of serious illness, ensuring that the treatment and care aligns with the patient’s wishes when autonomous decision-making becomes impossible. Notably, unlike merely signing legal documents, ACP focuses more on open communication (3).

In mainland China, Shenzhen became the first region to legislate living wills in 2022 (Article 78 of the Shenzhen Special Economic Zone Medical Regulations). However, palliative care legislation remains underdeveloped (4). In recent years, in response to the advocacy of “active aging” of Chinese government alongside the family-centric culture and immature legal framework in China, scholars have increasingly shifted their attention toward the communication process of ACP, instead of emphasizing the importance of written documents (5).

According to a recent prediction (6), China is expected to enter a phase of deep population aging by 2050, when the older adult population will reach 34.9% of the total population. China experiences a growing burden of chronic diseases in the older adult people. Despite ongoing cultural and legal challenges, ACP is anticipated to become an important component of future healthcare, as it not only enhances quality of life and preserves patient autonomy at the end stage of hospitalization, but also alleviates economic burdens and the decision-making stress for family members (7, 8).

With increasing age-related health risks, studies have shown that ACP could improve the quality of end-of-life care (9). ACP readiness reflects how prepared patients are to discuss serious illness care with physicians and family members, and it also predicts their likelihood of future ACP participation (10). Evidence suggests that family support plays a key role in both initiating and sustaining ACP (11, 12). Emotional support, in particular, mitigates fear and resistance, making it easier for patients to discuss ACP with their families. Older adult individuals often encounter declining physical function and increased social isolation due to changing social roles. Relevant studies (13, 14) have shown that strong family support significantly enhances the physical and psychological well-being of older adult patients with chronic diseases by providing emotional and financial help. However, the relationship between quality of life and ACP readiness remains contested. While Wang Xinru et al. (10) reported that better physical and mental health positively influences ACP readiness, Li Yalin et al. (15) found that older adult patients with limited mobility are actually more receptive to ACP.

This study is the first to explore the interrelationships among family support, quality of life, and ACP readiness in older adult patients with chronic diseases. It provides novel insights based on China’s family-centered culture and medical decision-making traditions.

In short, this study examines one key idea: quality of life might explain how family support affects ACP readiness in older adult patients with chronic diseases. The results are presented below.

## 2 Subjects and methods

### 2.1 Subjects

Using convenience sampling, 262 older adult patients with chronic diseases were recruited from seven tertiary comprehensive hospitals across Guangdong, Hainan, Sichuan, Heilongjiang, and Hubei provinces between November 2024 and February 2025.

### 2.2 Inclusion criteria

1 Age ≥ 60 years old.
2 Clinically diagnosed with a confirmed chronic disease in the stable or recovery phase (including cardiovascular diseases, endocrine diseases, cerebrovascular diseases, chronic respiratory diseases, digestive system diseases, malignant tumors, etc.).
3 Clear consciousness with adequate comprehension and communication abilities.
4 Provided informed consent and voluntarily agreed to participate in this survey.

### 2.3 Exclusion criteria

1 Individuals with diagnosed cognitive or psychiatric disorders.
2 Patients in critical condition who are unable to cooperate with the questionnaire survey.

The sample size should be 10–15 times of the independent variables. Given that the study involved 13 independent variables and required structural equation modeling (SEM), a minimum of 200 participants was necessary. Accounting for a potential 15% invalidity rate, this study finally collects 262 valid questionnaires. This study has been approved by the Ethics Committee of Tongji Medical College, Huazhong University of Science and Technology (Approval No. [2022]-Ethical Review (S111)).

### 2.4 Methodology

#### 2.4.1 Research tools

(i) General information questionnaire

This questionnaire was self-developed based on a comprehensive literature review and expert consultation. It collected demographic and background information, including gender, age, education, marital status, place of residence, religious beliefs, per capita monthly household income, type of health insurance, number of chronic diseases, duration of chronic disease (years), experience of caring for a dying family member, and prior experience in making medical decisions on behalf of others.

(ii) Advance care planning (ACP) readiness scale

This scale was developed by Chinese scholar Wang Xinru et al. (16) to reflect China’s cultural context, and has been widely used among older adult chronic disease patients with good reliability and validity. It comprises 22 items across 3 dimensions: attitudes toward ACP (10 entries), beliefs about participating in ACP (5 entries), and motivation to participate in ACP (7 entries). Each entry was rated on a 5-point Likert scale. There were five options, ranging from “strongly disagree” to “strongly agree,” with scores ranging from 1 to 5, respectively. Attitudes toward ACP were scored inversely. The total score ranged from 22 to 110. Higher scores indicate better readiness for ACP. The Cronbach’s ɑ coefficient for this scale in this study was 0.882. The Cronbach’s ɑ coefficients for the three dimensions of attitude toward ACP, belief in participating in ACP, and motivation for participating in ACP were 0.844, 0.809, and 0.822, respectively.

(iii) EuroQol five-dimension five-level scale (EQ-5D-5L)

Developed by the EuroQol Group (17), the EQ-5D-5L scale was used in this study for health status assessment. The health status assessment system includes five single-item dimensions: mobility, self-care, usual activities, pain/discomfort, and anxiety/depression. Each dimension has five levels: no problems, slight problems, moderate problems, severe problems, and extreme problems (18, 19). Based on the utility value scoring system for the Chinese population, the health utility values measured by this scale range from −0.391 to 1.000 (18). In this study, the Cronbach’s *α* coefficient for the health status assessment system was 0.902.

(iv) Perceived social support from family scale, PSS-Fa

Developed by Procidano et al. (20), this scale has been widely used. It consists of 15 items, with “Yes” scored as 1 and “No” scored as 0. Items 3, 13, and 15 are reversely scored. The total score ranges from 0 to 15, with higher scores indicating better family support. The Kuder–Richardson 21 value for this scale was 0.75.

#### 2.4.2 Framework for mediation effect analysis

Based on the results provided by previous literature studies, we make the following assumptions: Family support can directly affect the ACP readiness of older adult patients with chronic diseases, and it can also indirectly affect their ACP readiness by influencing the quality of life of older adult patients. The preliminary construction of the mediating effect model analysis framework is as follows:

Independent variable (X): Family support.
Mediating variable (M): Quality of life.
Dependent variable (Y): Advance care planning readiness.

#### 2.4.3 Data collection methods

Before distributing the questionnaires, we contacted the nursing departments of all seven participating hospitals to obtain approval and support from their administrators. Each hospital designated one coordinator and one investigator (who did not participate in data analysis or evaluation). First, we conducted a unified online training session on ACP-related knowledge for the seven coordinators responsible for the questionnaire survey.

The investigators then visited clinical departments and used standardized instructions to explain the research purpose and the meaning of ACP to each eligible older adult patient with chronic diseases at their bedside. After obtaining written informed consent, they administered the questionnaires face-to-face and collected them immediately. The investigators verified the completeness of each questionnaire on-site and prompted participants to complete any missing items to ensure data quality. For patients who had difficulty in completing the questionnaire independently, the investigators recorded their responses verbatim based on oral input.

A total of 300 questionnaires were distributed. After excluding those with patterned responses or logical inconsistencies, 262 valid questionnaires were ultimately collected, yielding an effective response rate of 87.3%.

#### 2.4.4 Statistical methods

SPSS 26.0 software was used for data entry and analysis. Categorical variables were described as frequencies and percentages. The Kolmogorov–Smirnov test was used to assess the normality of the data. Normally distributed continuous variables were expressed as mean ± standard deviation (x̄ ± s), while non-normally distributed data were presented as median (*P*_25_, *P*_75_). Spearman correlation analysis was performed to evaluate associations between variables.

Structural equation modeling (SEM) was conducted using AMOS 24.0 for path analysis, and the Bootstrap method was applied to test for mediating effects. The significance level (*α*) was set at 0.05.

## 3 Results

### 3.1 Demographic characteristics of older adult patients with chronic diseases

A total of 262 older adult patients with chronic diseases participated in the study, including 119 males (45.4%) and 143 females (54.6%). Detailed information is presented in Table 1.

**Table 1:** Demographic data of older adult patients with chronic diseases (*n* = 262).

Variables	Classification	Sample size	Percentage (%)
Gender	Male	119	45.4
	Female	143	54.6
Age groups	60 ~ 69	107	40.8
	70 ~ 79	97	37.0
	80 and above	58	22.1
Educational level	Illiterate	19	7.3
	Primary school	68	26.0
	Secondary school	59	22.5
	High school	68	26.0
	College and above	48	18.3
Marital status	Married	213	81.3
	Divorced/Widowed	49	18.7
Religion	Have	46	17.6
	None	216	82.4
Number of children	One and less	82	31.3
	Two	129	49.2
	Three	51	19.5
Residence	Town	170	64.9
	Village	92	35.1
Household per capita monthly income	<1,000	28	10.7
	1,000 ~ 3,000	64	24.4
	3,000 ~ 6,000	84	32.1
	6,000 above	86	32.8
Medical insurance	Self-paid	16	6.1
	Urban and rural resident basic	128	48.9
	Urban employee basic	82	31.3
	Compulsory	36	13.7
The number of chronic diseases	One	112	42.7
	Two	85	32.4
	Three	30	11.5
	Three and more	35	13.4
Duration of suffering from chronic diseases	<5 years	111	42.4
	5 ~ 10 years	87	33.2
	>10 years	64	24.4
Once caring for the families of the terminally ill	Yes	129	49.2
	No	133	50.8
Have made medical decisions for others	Yes	82	31.3
	No	180	68.7

### 3.2 Scores of ACP readiness, quality of life, and family support in older adult patients with chronic diseases

The ACP readiness score of older adult patients with chronic diseases was 81.97 ± 9.88, indicating an above-average level. The family support score was 12.00 (12.00, 13.00), reflecting a relatively high level of family support. The score of quality of life was 0.831 (0.640, 0.942), also indicating an above-average level. Specific indicators are detailed in Table 2.

**Table 2:** Scores of ACP readiness, quality of life, and family support in older adult patients with chronic diseases (*n* = 262).

Variables	Scores *M* (*P*_25_, *P*_75_)	Item average score *M* (*P*_25_, *P*_75_)
ACP readiness	81.97 ± 9.88^a^	3.73 ± 0.45^a^
Attitude dimension	36.00 (30.00, 40.00)	3.60 (3.00, 4.00)
Belief dimension	20.00 (18.00, 21.00)	4.00 (3.60, 4.20)
Motivation dimension	27.50 (26.00, 30.00)	3.93 (3.71, 4.29)
Family support	12.00 (12.00, 13.00)	0.80 (0.80, 0.87)
Quality of life	0.831 (0.640, 0.942)	—
Mobility	0.033 (0.000, 0.066)	0.033 (0.000, 0.066)
Self-care	0.000 (0.000, 0.048)	0.000 (0.000, 0.048)
Usual activities	0.000 (0.000, 0.045)	0.000 (0.000, 0.045)
Pain or discomfort	0.058 (0.000, 0.138)	0.058 (0.000, 0.138)
Anxiety or depression	0.025 (0.000, 0.049)	0.025 (0.000, 0.049)

^a^ indicates that the data conforms to a normal distribution.

### 3.3 Correlation analysis of ACP readiness, family support, and quality of life in older adult patients with chronic diseases

Family support was positively correlated with both ACP readiness and quality of life (*r* = 0.515, 0.403, respectively; both *p* < 0.01). In addition, quality of life was positively associated with ACP readiness (*r* = 0.552, *p* < 0.01). Specific indicators are detailed in Table 3.

**Table 3:** Correlation analysis of ACP readiness, family support, and quality of life in older adult patients with chronic diseases (*n* = 262).

Variables	Correlation coefficient		
	ACP readiness	Family support	Quality of life
ACP readiness	1		
Family support	0.515**	1	
Quality of life	0.552**	0.403**	1

**p<0.01.

### 3.4 Analysis of the mediating effect of quality of life on the relationship between family support and advance care planning (ACP) readiness in older adult patients with chronic diseases

#### 3.4.1 Common method bias test

A Harman’s single-factor test was performed to assess the items of the three scales in this study. A total of 11 common factors with eigenvalues > 1 were extracted. The first factor without rotation explained 22.66% of the total variance, which is below the critical threshold of 40%. This indicates that there is no significant common method bias in this study.

#### 3.4.2 Mediating effect test

**Figure 1:**
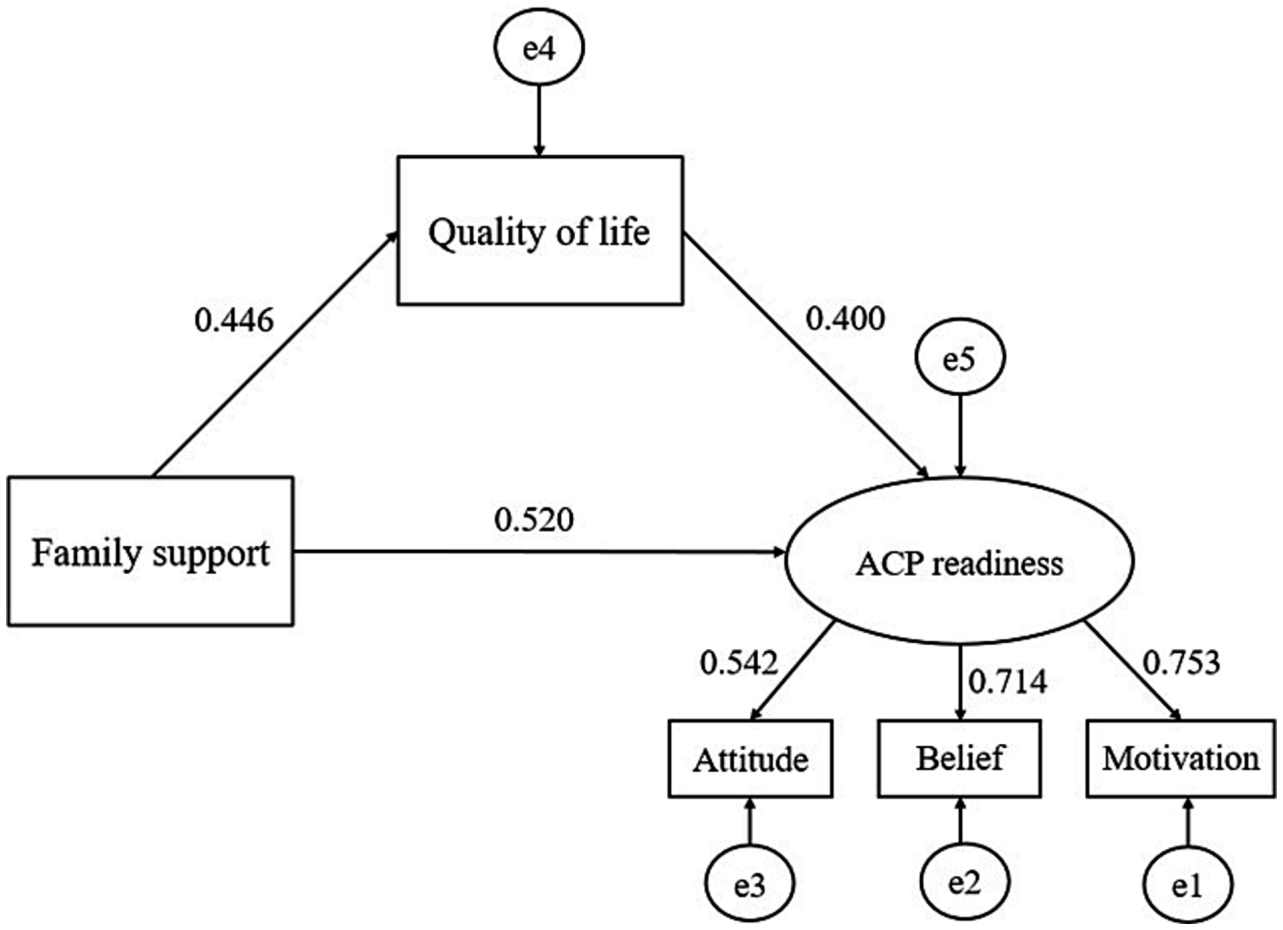
Path diagram of family support, quality of life, and ACP readiness. The numerical results presented in the figure are all standardized coefficients.

Structural equation modeling was constructed using AMOS24.0 software, with family support as the independent variable, quality of life as the mediating variable, and ACP readiness as the dependent variable, as shown in Figure 1. The results showed: χ^2^/df = 1.015, RMSEA = 0.008, CFI = 1.000, GFI = 0.994, NFI = 0.989, IFI = 1.000, TLI = 1.000. According to the model fit criteria proposed by Fang Jie et al. (21), these indicators are within acceptable ranges, indicating that the model is acceptable. According to the results of structural equation modeling, family support positively predicted quality of life (*β* = 0.446, *p* < 0.001); family support positively predicted ACP readiness (*β* = 0.520, *p* < 0.001); and quality of life positively predicted ACP readiness (*β* = 0.400, *p* < 0.001) in older adult patients with chronic diseases. The Bootstrap method was used to test the mediating effect of the model, with 2000 random samples and a 95% confidence interval. The results showed that the confidence intervals for the total, direct, and indirect effects of family support on ACP readiness did not include 0, indicating that quality of life partially mediates the relationship between family support and ACP readiness. The mediating effect accounted for 25.5% of the total effect, as presented in Table 4.

**Table 4:** Mediating effect of quality of life on the relationship between family support and ACP readiness.

Variables	Effect	SE	95% CI	*p*	Effect proportion (%)
Overall effect	0.698	0.047	0.729 ~ 1.079	<0.001	
Direct effect	0.520	0.084	0.511 ~ 0.847	0.001	74.50%
Indirect effect	0.178	0.091	0.154 ~ 0.345	0.001	25.50%

SE, bootstrap standard error; 95% CI, the lowest level of the 95% Bootstrap confidence interval ~ the highest level of the 95% Bootstrap confidence interval.

## 4 Discussion

In this study, the score of quality of life, as measured by the EQ-5D-5L utility index, was 0.831 (0.640, 0.942), which is higher than the 0.72 ± 0.24 reported in a study of older adult Vietnamese patients with chronic diseases (22). Based on the mean scores across the five EQ-5D-5L dimensions (mobility, self-care, usual activities, pain/discomfort, and anxiety/depression), most participants demonstrated good physical function and emotional well-being. They were largely capable of performing daily activities independently and exhibited positive mental states without severe anxiety or depression. These outcomes may reflect the positive impact of national healthcare policies in China, such as chronic disease management programs and older adult welfare subsidies. Such measures alleviate the financial burden of medical expenses for older patients with chronic conditions, thereby preventing disease progression due to lack of access to treatment.

The family support score was 12.00 (12.00, 13.00), which is lower than scores reported among nursing home residents (13.49 ± 2.06) (23) and stroke patients [13.00 (12.00, 15.00)] (24). This may be attributed to factors such as reduced family caregiving due to institutional support and caregiver fatigue associated with prolonged illness duration. Previous quantitative studies (25, 26) have shown that ACP interventions increase family involvement in decision-making and also enhance concordance between patient preferences and family decisions.

The ACP readiness score in this study was 81.97 ± 9.88, indicating a moderately high level of readiness. This value is lower than that of chronic disease patients in Beijing communities (87.48 ± 12.96) (10), but higher than community-dwelling older adult chronic patients in Shanxi Province (75.21 ± 13.87) (15). During the process of data collection, investigators observed that most older adult chronic disease patients were unfamiliar with the concept of ACP, although some had already engaged in ACP-related practices in daily life (27). The present findings suggest that patients exhibited relatively positive attitudes, clear understanding, and strong motivation toward ACP, consistent with previous research by Wang et al. (10). These results may be explained by ongoing healthcare reform in China, which emphasizes value-based care and patient-centered approaches. Older adult individuals are increasingly prioritizing quality of life over longevity, reflecting a desire for “death with dignity” (28). Therefore, ACP attitudes become more positive. Based on the Health Belief Model (29), outcome expectations influence behavioral beliefs. When first introduced to ACP, patients are more likely to perceive its advantages rather than barriers, thereby reinforcing positive behavioral beliefs. And these beliefs then promote positive attitudes and intentions. These findings underscore the need for policymakers and healthcare institutions to improve public awareness of ACP. Through enhanced legislation, community-based health education, and the use of accessible media tools such as short videos, ACP could be broadly implemented in healthcare settings.

This study further confirmed that both family support and quality of life directly and positively influence ACP readiness (*p* < 0.01). According to Motivation-Behavior Theory, positive family support can enhance a patient’s intrinsic motivation, increasing their willingness to engage in advance care planning (30, 31). However, simply increasing a patient’s desire for autonomous decision-making may not be sufficient, research suggests that family engagement remains essential for successful ACP implementation (32). In fact, studies have shown that patients worldwide value family opinions when making decisions (9), and good family support provides emotional, economic, informational, and psychological assistance (11), which in turn reduces negative emotions and increases patients’ sense of dignity and security. Consequently, patients become more willing to discuss medical plans with families.

The mediation analysis in this study revealed that quality of life accounted for 25.5% of the mediating effect of family support on ACP readiness. This finding highlights a potential mechanism by which family support facilitates ACP readiness. A Delphi expert consensus identified “care consistent with patient wishes and goals” as ACP’s most important outcome (33). Previous researches (34, 35) have shown that positive family functioning correlates with better physical, mental, and social health. Family is vital for older adult well-being. Care and support among family members effectively improve their health status and quality of life (36). Although the effect of quality of life on ACP readiness was moderate (*β* = 0.400), it reveals that patients with better perceived health are more proactive in planning for future medical decisions. These results support the recommendation that ACP discussions should start during disease stable stage, and underscore the central role of family systems in palliative care. Families not only provide direct care but also serve as the psychosocial catalyst for initiating ACP.

Therefore, it is recommended that healthcare providers should pay close attention to the interrelationships among family support, quality of life, and ACP readiness in older adult patients with chronic diseases. Emphasis should be placed on assessing family dynamics and enhancing communication between patients and their families. Additionally, routine mental health screening and the establishment of comprehensive geriatric assessment system could allow more precise monitoring and recording of changes in patients’ quality of life, providing support for healthcare professionals to choose the most appropriate timing for interventions. These strategies would support timely ACP interventions, helping patients to make medical decisions that align with their own wishes, while avoiding unnecessary or burdensome treatment.

### 4.1 Limitations

This study has several limitations. First, the cross-sectional study cannot establish causality. Future longitudinal studies should incorporate multiple follow-up time points to track the dynamic relationships between family support, quality of life, and ACP readiness over time. Second, participants were recruited using convenience sampling from seven tertiary hospitals in five provinces. This introduces regional and institutional bias, as patients from primary care institutions, community settings, and rural areas were not represented. As a result, the generalizability of the findings is limited. Future research should adopt a multi-center sampling approach, including participants from community health centers, senior care facilities, and rural populations, to allow for comparative analyses across geographic and healthcare system contexts. Third, other potentially relevant variables were not assessed and warrant further exploration. Despite these limitations, this study provides the first empirical investigation into the relationships among family support, quality of life, and ACP readiness in older adult patients with chronic diseases. It offers a unique perspective grounded in China’s cultural and healthcare context, particularly within the physician-patient-family triadic decision-making model.

## 5 Conclusion

This study demonstrates that the ACP readiness of older adult patients with chronic diseases is at a moderately high level. Family support can directly enhance their ACP readiness, and it can also indirectly influence ACP readiness through quality of life. The findings provide valuable insights into the underlying mechanisms that shape ACP readiness in older adult population. They also suggest that targeted interventions informed by these results could be developed to further improve ACP readiness among older adult patients with chronic diseases.

## Data Availability

The raw data supporting the conclusions of this article will be made available by the authors, without undue reservation.
